# Hypothermia versus normothermia after out-of-hospital cardiac arrest: A systematic review and meta-analysis of randomized controlled trials

**DOI:** 10.1016/j.amsu.2022.103327

**Published:** 2022-01-29

**Authors:** Dhan Bahadur Shrestha, Yub Raj Sedhai, Pravash Budhathoki, Suman Gaire, Anurag Adhikari, Ayusha Poudel, Barun Babu Aryal, Wasey Ali Yadullahi Mir, Khagendra Dahal, Markos G. Kashiouris

**Affiliations:** a Department of Internal Medicine, Mount Sinai Hospital, Chicago, IL, USA; b Department of Internal Medicine, Division of Hospital Medicine, Virginia Commonwealth University, School of Medicine, Richmond, VA, USA; c Department of Internal Medicine, Bronxcare Health System, Bronx, NY, USA; d Department of Emergency Medicine, Palpa Hospital, Palpa, Nepal; e Department of Emergency Medicine, Nepal National Hospital, Kathmandu, Nepal; f Department of Emergency Medicine, Alka Hospital, Kathmandu, Nepal; g Nepalese Army Institute of Health Sciences, Kathmandu, Nepal; h Department of Internal Medicine, Division of Cardiology, Creighton University School of Medicine, Omaha, NE, USA; i Department of Internal Medicine, Division of Pulmonary Disease and Critical Care Medicine, VCU School of Medicine, Richmond, VA, USA

**Keywords:** Out-of-hospital cardiac arrest, Temperature, Induced Hypothermia, OHCA, Out-of-hospital cardiac arrest, TTM, Targeted temperature management, IHCA, In-hospital cardiac arrest, ROSC, Return of spontaneous circulation, PRISMA, Preferred Reporting Items for Systematic Review and Meta-Analysis, RCTs, Randomized controlled trials, TH, Therapeutic hypothermia, AHA, American Heart Association, ESC, European resuscitation council, OR, Odds ratio, CI, Confidence interval

## Abstract

**Background:**

The current guidelines recommend targeted temperature management (TTM) as part of the post-resuscitation care for comatose patients following out-of-hospital cardiac arrest. These recommendations are based on the weak evidence of benefit seen in the early clinical trials. Recent large multicentered trials have failed to show a meaningful clinical benefit of hypothermia, unlike the earlier studies. Thus, to fully appraise the available data, we sought to perform this systematic review and meta-analysis of randomized controlled trials.

**Methods:**

We searched four databases for randomized controlled trials comparing therapeutic hypothermia (32–34 °C) with normothermia (≥36 °C with control of fever) in adult patients resuscitated after out-of-hospital cardiac arrest. Independent reviewers did the title and abstract screening, full-text screening, and extraction. The primary outcome was mortality six months after cardiac arrest, and secondary outcomes were neurological outcomes and adverse effects.

**Relevance for patients:**

Six randomized controlled trials were included in this review. There was no significant difference between the hypothermia and normothermia groups in mortality till 6 months follow up after out-of-hospital cardiac arrest (OR 0.88, 95% CI 0.67–1.16; n = 3243; I^2^ = 51%), or favorable neurological outcome (OR 1.31, 95% CI 0.93–1.84; n = 3091; I^2^ = 68%). Rates of arrhythmias were notably higher in the hypothermia group than the normothermia group (OR 1.43, 95% CI 1.20–1.71; n = 3029; I^2^ = 4%). However, odds for development of pneumonia showed no significant differences across two groups (OR 1.13, 95% CI 0.98–1.31; n = 3056; I^2^ = 22%). Therefore, targeted hypothermia with a target temperature of 32–34 °C does not provide mortality benefit or better neurological outcome in patients resuscitated after the out-of-hospital cardiac arrest when compared with normothermia.

## Highlights

• Targeted hypothermia did not provide significant differences in mortality after OHCA (OR 0.88, 95% CI 0.67–1.16).
• Similarly, neurological outcome did not show significant differences between two arms (OR 1.31, 95% CI 0.93–1.84).
• Arrhythmias were more in the hypothermia group than normothermia group (OR 1.43, 95% CI 1.20–1.71).

## 1 Introduction

In the United States, more than 292,000 adults experience cardiac arrest every year [1]. The mean survival of out-of-hospital cardiac arrest (OHCA) is approximately 10% [2], and the mean survival in-hospital cardiac arrest (IHCA) is approximately 25% [3]. Cardiac arrest survivors have a substantial risk of neurological injury due to hypoxia, ischemia, reperfusion injury, and excitotoxicity [4]. The current American heart association (AHA) guideline recommends targeted temperature management (TTM) between 32 °C and 36 °C for at least 24 hours for patients unresponsive after OHCA and IHCA for all cardiac rhythms (Class I) [5]. The European resuscitation council (ESC) also recommends TTM with a target temperature at a constant value between 32 and 36 °C for at least 24 hours for adults who remain unresponsive after the return of spontaneous circulation (ROSC) in either OHCA or IHCA with any initial rhythm [6]. These recommendations are largely based on the weak evidence of benefit, mostly from earlier clinical trials with many limitations [7,8]. Recently, the most recent targeted temperature management-2 (TTM-2) trial randomized 1861 patients to the targeted hypothermia (33 °C) or targeted normothermia (37.8 °C) groups and found no benefit of targeted hypothermia in reducing mortality at six months [9]. Also, the TTM-2 trial showed no difference in survival with severe neurological disability and instead showed a higher risk of arrhythmias among hypothermia-treated patients, contradicting the prior studies. Thus, to fully appraise the available data, we sought to perform this systematic review and meta-analysis of randomized controlled trials. Herein we have included randomized controlled trials that compared targeted hypothermia (32–34 °C) with normothermia (≥36 °C with control of fever).

## 2 Methods

Our study followed the Preferred Reporting Items for Systematic Review and Meta-Analysis (PRISMA) guidelines [10]. In addition, the study protocol has been registered in the International prospective register of systematic reviews (PROSPERO ID: CRD42021268800) [11].

### 2.1 Criteria for considering studies for this review

#### 2.1.1 Type of studies

Randomized controlled trials (RCTs) comparing targeted hypothermia and normothermia after out-of-hospital cardiac arrest and reporting any of the outcomes of interest, including mortality, functional and neurological outcomes, length of hospitalization, and adverse effects, were included in the review. Unpublished, non-randomized, or non-experimental studies were excluded.

#### 2.1.2 Type of participants

Patients above 18 who had a return of spontaneous circulation after out of hospital cardiac arrest with any cardiac rhythm regardless of the underlying etiology or comorbidities were included.

#### 2.1.3 Types of interventions

Studies were included if the patients were randomized to two groups: an intervention group (either pre-hospital or in-hospital targeted hypothermia of≤35 °C) and the control group (normothermia (>35 °C and <37.5 °C). Studies comparing pre-hospital cooling with intrahospital cooling where the controls were maintained in normothermia in the pre-hospital setting and were subjected to hypothermia in the hospital setting were excluded from our analysis.

#### 2.1.4 Types of outcome measures

Studies that reported any of the outcomes of interest (mortality, functional or neurological outcomes, and adverse effects) were included in the analysis. No timeframes were set for follow-up or reporting outcomes or the indices used to assess functional and neurological outcomes.

### 2.2 Outcomes

The primary outcomes of interest were in-hospital mortality and mortality at six months. Secondary outcomes of interest were the length of hospitalization, functional outcome, and adverse events. In addition, neurological outcomes assessed with either Cerebral Performance Category (CPC) score or Modified Rankin Scale (mRS) were analyzed. Apart from the clinical outcomes, baseline characteristics of patients on admission, including age, sex, site of cardiac arrest, inclusion and exclusion criteria of the particular studies, were also included.

### 2.3 Search methods for identification of studies

We searched the PubMed, PubMed Central (PMC), Scopus, and Embase databases and included relevant randomized control trials published between January 1, 2000, and July 31, 2021. We ran the search using MeSH terms which included targeted temperature management, targeted hypothermia, targeted normothermia, cardiac arrest, and their commonly used synonyms. In addition, articles published in English and articles published in other languages which had English translation available online were included.

#### 2.3.1 Electronic searches

The detailed search strategy has been attached in Supplementary material 1.

### 2.4 Data collection and analysis

Covidence systematic review software was used to screen studies and extract data. Cochrane Review Manager (RevMan) version 5.4 was used for the data analysis.

### 2.5 Selection of studies

All studies were first screened based on their titles and abstracts by two independent reviewers using the Covidence systematic review software. Next, a third reviewer resolved the conflicts. Full texts of the selected studies were then further screened following the same method. Data were then extracted from all studies selected via full-text review for qualitative and quantitative analysis. Finally, another independent reviewer assessed the risk of bias and cross-checked the selected studies.

### 2.6 Data extraction and management

Three researchers independently extracted the data, and it was verified in the presence of a fourth reviewer. Data extracted included the study details, including the inclusion and exclusion criteria, demographic and baseline characteristics of the patients, reported intervention and comparison groups, and the outcomes of interest. Any disagreements were resolved by discussion among the four reviewers. In cases of missing or incongruent data or need for additional details, the study's authors requested other data or clarification. Data was recorded in Covidence and later exported to Cochrane Review Manager (RevMan) version 5.4 for analysis. Outcomes were measured using a fixed or random effect model for dichotomous variables and the mean difference for continuous variables.

### 2.7 Assessment of risk of bias in included studies

**Fig. 1:**
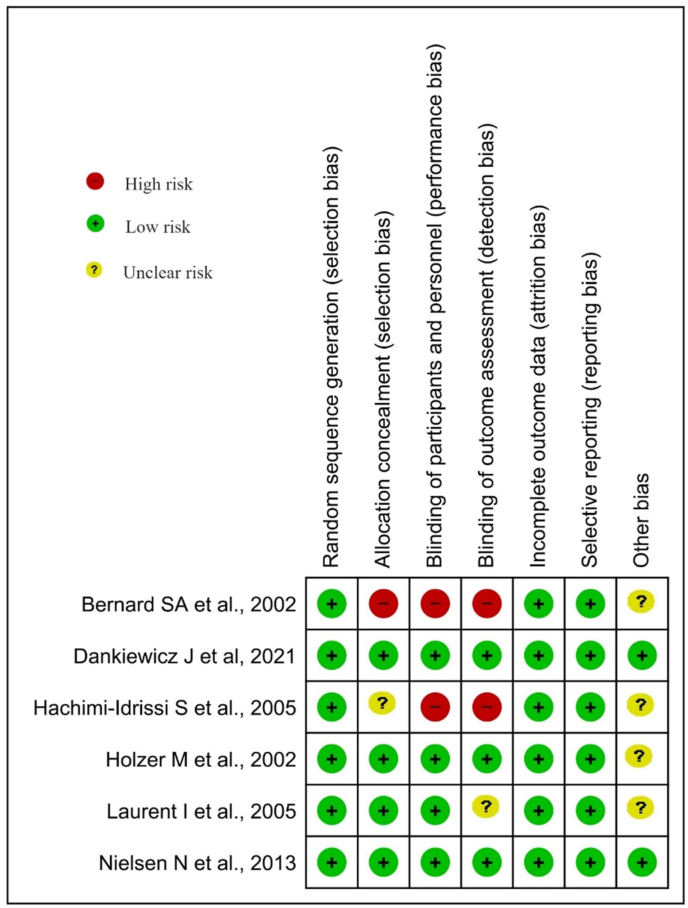
Risk of Bias assessment across RCTs.

Cochrane RoB 2.0 was used to assess the risk of bias in the included studies (Fig. 1) [12].

### 2.8 Assessment of heterogeneity

The heterogeneity in the included studies was determined using the I^2^ test using the Cochrane Handbook for Systematic Reviews of Interventions [13].

### 2.9 Assessment of reporting biases

Reporting bias was checked by prefixed reporting of the outcome.

### 2.10 Data synthesis

RevMan 5.4 was used to perform statistical analysis. Outcome estimation was done using Odds Ratio (OR) with a 95% confidence interval.

### 2.11 Investigation of heterogeneity

Heterogeneity was assessed using I-square (I^2^) test; the fixed/random effects model were used based on heterogeneity.

## 3 Results

**Fig. 2:**
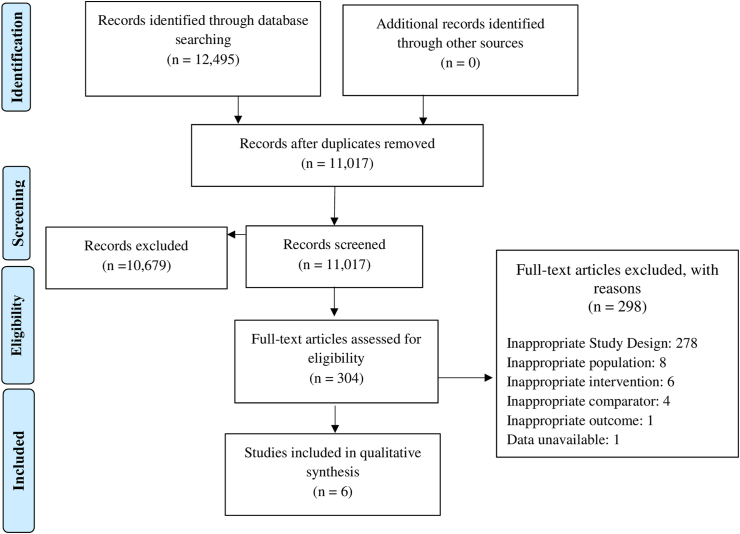
PRISMA flow diagram.

Twelve thousand four hundred ninety-five studies were identified by the database searches, out of which 11017 underwent title and abstract screening after removal of duplicates. The full texts of 304 studies were then assessed for eligibility. Seven studies were determined to be eligible and included in the analysis. Fig. 2 shows the PRISMA flow diagram of our study.

We included six randomized controlled trials with 3255 patients [[7], [8], [9],[14], [15], [16]]. Thus, our study has only out of hospital cardiac arrests (OHCAs) in which hypothermia was compared to normothermia. The characteristics of included trials are summarized in Table 1, and basic details are provided in Supplementary file 2.

**Table 1:** Narrative summary of included studies.

Study ID	N(T:C)	Age	Sex (M:F)	Intervention				Comparison	Outcomes	
				Target temperature	Time of commencement	Duration	Rewarming	Target temperature	Mortality	Unfavorable neurological outcome*
Bernard et al., 2002 [8]	77 (43:34)		52:25; T = 25:18; C = 27:7	33 °C	within 2 hours after ROSC	12 hours	Active rewarming after 18 hours	37 °C	T = 22/43; C = 23/34	T = 0/43; C = 2/34 (Severe disability: entirely dependent or unconscious)
Dankiewicz et al., 2021 [9]	1861 (930:931)	T = 64 + 13C = 63 ± 14	1477:384; T = 742:188; C = 735:196	33 °C	After randomization	28 hours	Rewarming after 28 hours	37.5 °C	T = 465/925; C = 446/925	T = 23/881; C = 47/866 (mRS 4 or 5)
Hachimi-Idrissi et al., 2004 [15]	61 (30:31)	SSP: T = 72.5 ± 3C = 74.1 ± 2; LSP 61.3 ± 2C = 62.7 ± 3	44:17; T = 23:7; C = 21:10	33 °C	within 60 min from collapse	24 hours	Passive rewarming over 8 hours	<38 degrees C	T = 18/30; C = 23/31	T = 4/30; C = 5/31 (CPC 3 or 4)
Hypothermia after Cardiac Arrest Study Group, 2002 [7]	275 (137:138)	Median (IQR): T = 59 (51–69); C = 59 (49–67)	210:65; T 104:33; C = 106:32	32–34 °C	After being brought to the ER	24 hours	Passive rewarming over 8 hours	‘normothermia'	T = 56/136; C = 76/138	T = 5/136; C = 9/138 (CPC 3 or 4)
Laurent et al., 2005 [16]	42 (22:20)	Median (IQR): T = 56 (50–70); C = 52 (47–59)	34:8; T = 18:4; C = 16:4	32 °C		16 hours after hemofiltration was stopped (24 hours after randomization)	after 24 hours	37 °C	At six months, T = 15/22; C = 11/20	
Nielsen et al., 2013 [14]	939 (473:466)	T = 64 ± 12; C = 64 ± 13	761:178; T = 393:80; C = 368:98	33 °C	at the time of randomization	36 hours	After 28 hours, gradual rewarming to 37 °C in increment of 0.5 per hour.	36 °C	T = 235/473; C = 225/466	T = 23/469; C = 22/464 (CPC 3 or 4)

N total number of patients, T patients in the intervention group, C patients in the control group, M male, F female, °C degrees Celsius, IQR interquartile range, ICU intensive care unit, SSP short study period, LSP long study period, CPC Cerebral performance category grade, VT ventricular tachycardia.

Bernard et al. performed an RCT in Australia, including patients with initial ventricular fibrillation managed with therapeutic hypothermia at 33 °C or normothermia at 37 °C [8]. A multinational RCT by Dankiewicz et al. compares OHCAs with presumed cardiac arrest, comparing a target temperature of 33 °C with a maintained temperature of 37.5 [9]. Hachimi-Idrissi et al. conducted an RCT in Belgium, primarily looking for astroglial S-100 b protein in patients with cardiac arrest treated with mild hypothermia. Here, we have compared the endpoints as provided between 33 °C and less than 38 °C [15]. In a multicenter RCT, Hypothermia after Cardiac Arrest Study Group reached hypothermia maintained between 32°C and 34 °C with maintained normothermia [7]. Laurent et al. performed an RCT in France to evaluate hemofiltration after out-of-hospital cardiac arrest. We assessed the outcomes between the hypothermia group at 32 °C and normothermia at 37 °C [16]. Nielsen et al., an RCT from Europe and Australia, compared hypothermia at 33 °C with normothermia at 36 °C [14].

### 3.1 Mortality till six months follow up

**Fig. 3:**
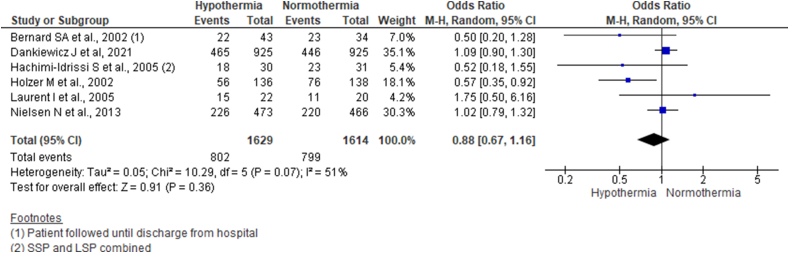
Forest plot comparing six-month mortality across hypothermia and normothermia protocol using a random-effect model.

We pooled the data using a random-effect model from six studies. It showed no significant differences in mortality at six months after OHCA (OR 0.88, 95% CI 0.67–1.16; n = 3243; I^2^ = 51%) (Fig. 3).

Subsequently, excluding studies with sample less than 50 in each arm (Bernard SA et al., Hachimi-Idrissi S et al., and Laurent I et al.) also showed a similar result (OR 0.93, 95% CI 0.70–1.23; n = 3063; I^2^ = 67%) (Supplementary file 3, Fig. 1). Additionally, excluding all the studies published before 2010 and pooling data from Nielsen N et al., and Dankiewicz J et al. using fixed effect model also showed no difference in mortality across two groups in 6 months following OHCA (OR 1.06, 95% CI 0.92–1.23; n = 2789; I^2^ = 0%) (Supplementary file 3, Fig. 2).

### 3.2 Favorable neurological outcomes

**Fig. 4:**
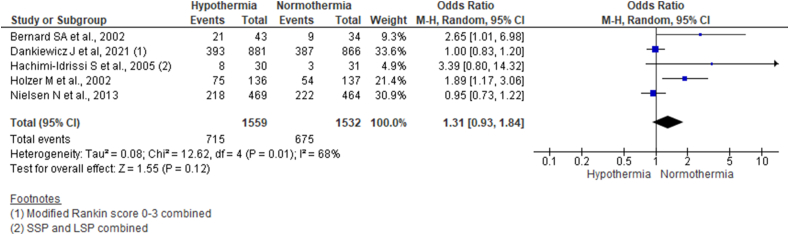
Forest plot comparing favorable neurological outcome six months following OHCA across hypothermia and normothermia protocol using a random-effect model.

Running analysis for favorable neurological outcome of CPC 1 or 2 or mRS of 0–3; the pooled data from five studies reporting neurological outcome using random-effect model did not show statistically significant differences between the two arms (OR 1.31, 95% CI 0.93–1.84; n = 3091; I^2^ = 68%) (Fig. 4).

Additionally, further running analysis for the unfavorable neurological outcome with CPC 3 or 4, or mRS of 4 or 5 using fixed effect model also did not show significant differences across the hypothermia and normothermia groups (OR 0.79, 95% CI 0.54–1.15; n = 2818; I^2^ = 0%) (Supplementary file 3, Fig. 3).

### 3.3 Arrhythmia and pneumonia

**Fig. 5:**
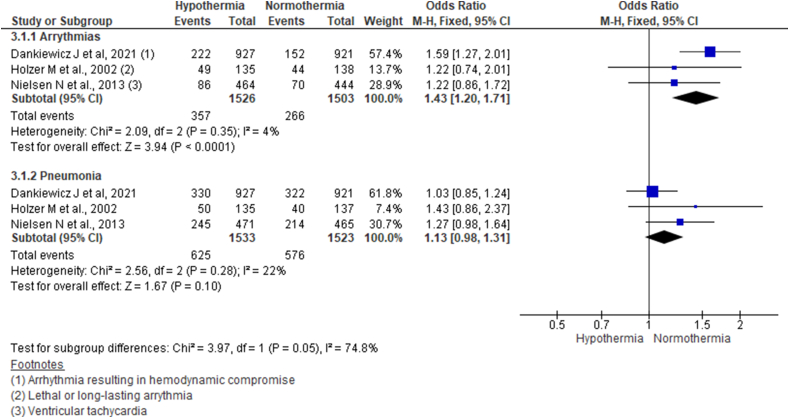
Forest plot comparing the occurrence of notable arrhythmias and pneumonia till six months following OHCA across hypothermia and normothermia protocol using fixed effect model.

Pooling data from three studies using the fixed-effect model showed notable arrhythmias more in the hypothermia group than normothermia group (OR 1.43, 95% CI 1.20–1.71; n = 3029; I^2^ = 4%). However, odds for development of pneumonia showed no significant differences across two groups (OR 1.13, 95% CI 0.98–1.31; n = 3056; I^2^ = 22%) (Fig. 5).

## 4 Discussion

In this meta-analysis, we compared clinical outcomes after out-of-hospital cardiac arrest (OHCA) managed with targeted hypothermia (32–34 °C) versus normothermia (≥36 °C with control of fever). Meta-analysis of pooled data from six randomized controlled trials showed no significant mortality difference between the two groups at six months. There were no significant differences in terms of favorable neurological outcomes. We observed a higher incidence of arrhythmia in the hypothermia group, with no significant differences in the incidence of pneumonia across to groups. The plausible mechanisms for the neuroprotective effect of therapeutic hypothermia (TH) include decreased cerebral metabolism, reduced generation of free radicals, cold-induced protein, anti-inflammatory effects, and decreased excitotoxic amino acids and neurotransmitters [[17], [18], [19]]. Furthermore, beneficial effects are seen in pre-clinical animal studies [20,21]. However, although initial landmark trials showed a clinical benefit, adequately powered more recent trials have shown contradicting results. Also, the earlier perceived evidence of benefit is further questioned by the evidence added by our meta-analysis.

The two landmark clinical trials published in 2002 showed therapeutic hypothermia to reduce neurological disability in patients with OHCA due to an initial shockable rhythm who remained comatose in the post-arrest period [7,8]. The Hypothermia after Cardiac Arrest Study (HACA trial) also showed a statistically significant mortality benefit in the hypothermia arm [7]. These trials led to the rapid adoption of therapeutic hypothermia in clinical practice. TH has received a class I recommendation for out-of-hospital cardiac arrest by both the American Heart Association (AHA) and European resuscitation council (ESC) guidelines since 2005 [22,23]. There was a subsequent expansion to include IHCA and non-shockable rhythms [24]. The current AHA and ESC resuscitation guidelines recommend TH as a Class I recommendation for unresponsive adults after achieving ROSC following an OHCA [5,6].

There are several limitations of the RCTs on TH that merit discussion. Both the initial landmark trials were small and included 352 patients when combined in both the studies [7,8]. The temperature was not actively managed in the normothermia arm, and a substantial number of patients in the control arm developed a fever. Thus, it is difficult to discern if the benefit seen was an actual effect of hypothermia or due to the deleterious effect of fever on neurological recovery in the normothermia group. Both the studies lacked blinding and a standardized protocol for neuro-prognostication. Thus, outcome data could have been affected by a premature withdrawal of care in the normothermia arm [7,8]. Over the last two decades, there has been a substantial evolution in critical care practices and post resuscitation care. Thus, the effect of TH seen in initial studies may not have replicated in the subsequent clinical trials. The more recent clinical trials have tried to address the limitations of the initial landmark trials. The TTM trial randomized 950 patients with an OHCA to a targeted temperature of 33 °C versus 36 °C, with active temperature management in both groups [14]. TTM trial showed no significant difference in mortality (50% vs. 48%; *P* = 0.51) or a composite of mortality and poor neurological outcome at six months (54% vs. 52%; *P* = 0.78) between hypothermia and the normothermia arms. Although smaller the findings of the FROST-I trial, a randomized multicenter pilot trial was consistent with the findings of the TTM trial [25]. It is important to note that non-shockable rhythms were under-represented in these trials, comprising only 20% in the TTM trial, while >75% of cardiac arrests are due to asystole or pulseless electrical activity. The HYPERION trial compared the effect of TH (33 °C) versus targeted normothermia (37 °C). It randomized 584 comatose patients after OHCA or IHCA due to an initial non-shockable rhythm. The trial showed a higher 90-day survival with favorable neurological outcomes in the TH group (10.2% vs. 5.7%; 95% [CI], 0.1 to 8.9, P = 0.04). However, the statistical finding was limited by a wide confidence interval [26]. We have not included the HYPERION trial in the meta-analysis given the inclusion of IHCA patients in the study.

The recently published TTM-2 trial included 1861 patients with OHCA at 61 sites across Australia, Europe, and the US who were comatose after OHCA. Patients were randomized to TH of 33 °C versus targeted normothermia (goal temperature: <37.5 °C). There were no differences in survival at six months (50% vs. 48%, P = 0.37) and survival with a severe disability on the modified Rankin scale (55% vs. 55%; RR 1.00; 95% CI, 0.92–1.09) across two groups. The findings of the TTM-2 trial contradict that of the HYPERION trial; however, they are consistent with the results of the TTM trial. TTM-2 trial is the largest study on targeted temperature management to date. Apart from randomization, both the groups were similarly treated in mechanical ventilation and neuromuscular blockade. Neurological prognostication was performed using a standard protocol, and withdrawal of care before 96 hours was discouraged.

Three recent meta-analyses have been published since the publication of the TTM 2 trial [[27], [28], [29]]. Fernando et al. compared the clinical outcomes with the degree of hypothermia. There was no difference between the two groups in deep hypothermia (31–32 °C), moderate hypothermia (33–34 °C), or mild hypothermia (35–36 °C) [29]. In the meta-analysis by Sanfilippo et al., hypothermia did provide benefits in terms of survival and neurological outcome compared to uncontrolled normothermia [27]. However, the benefits did not hold compared to cases with controlled normothermia with avoidance of fever. It is important to note that all the prior studies have smaller sample sizes. Hence, TTM and TTM 2 trials have larger weights in the analysis affecting the results of meta-analyses, including ours.

TTM 2 trial has reported arrhythmia causing hemodynamic compromise, bleeding, sepsis, pneumonia, and skin complications arising from devices for temperature management as to complications in their trial. We pooled the data for arrhythmia and pneumonia from the included studies because of inconsistencies in reporting the other outcomes. Among them, only the odds of arrhythmia were found to be higher in the hypothermia group. The cause of increased arrhythmia seems to be multifactorial, including the direct effects of hypothermia on the myocardium [30].

There are several strengths of our meta-analysis. First, the study was conducted as per a pre-registered protocol. Second, a thorough literature search is performed using multiple databases. Third, only RCTs that have included out-of-hospital cardiac arrest have been included in the analysis. There are many factors to be considered while interpreting the results of our meta-analysis. We have included studies performed over two decades. There has been substantial evolution in post-resuscitation care from early 2000 to recent times. Additionally, the availability of public defibrillators, bystander CPR, and response time of emergency medical services have changed over the years affecting the clinical outcomes. E.g., >90% of cardiac arrests were witnessed, and approximately 80% received bystander cardiopulmonary resuscitation (CPR) in the TTM 2 trial. Various patient-related factors like gender, age, etiology, pre-existing comorbidity can also affect the clinical outcomes after cardiac arrest [31]. Contrasting clinical outcomes in the later trials, including the TTM 2, could be attributed to these factors. Cumulatively results of our meta-analysis reflect the impact of these factors and add to the granularity of the data.

Our study has some limitations. The included studies have their inherent limitations. Active cooling was performed in 46% of patients in the normothermia group in the TTM 2 study. This highlights the higher prevalence of fever and underscores the importance of temperature management. We could not perform subgroup analysis between surface and intravascular cooling devices. The mean duration of the initial contact time to randomization in the TTM 2 trial was 136 minutes. Also, the median time to achieve the target temperature of 34 °C was 3 hours after randomization. This amounts to a total duration of 5 h to achieve the target temperature. It remains to be seen if a more rapid cooling rate would have demonstrated a clinical benefit of TH. Although prior studies on pre-hospital cooling in OHCA have failed to show a mortality benefit [32,33], future clinical trials may discern if more rapid cooling results in better neuroprotection.

## 5 Conclusion

Based on the results of our meta-analysis, therapeutic hypothermia with a targeted temperature of 32–34 °C does not reduce mortality at six months among comatose patients after OHCA. There were no significant differences across the two groups regarding favorable neurological outcomes. We observed a higher incidence of arrhythmia in the hypothermia group. The current resuscitation guidelines recommend TTM for comatose patients after OHCA. However, given the lack of benefit in the available data and limitations of the existing. Studies support the need for additional evidence for TTM in OHCA patients.

## Ethics approval and consent to participate

Not applicable.

## Sources of funding

This article did not receive any specific grant from funding agencies in the public, commercial, or other sectors.

## Authors' contributions

DBS, YRS, and KD contributed to the concept and design, analysis, and interpretation of data. DBS, PB, SG, AA, AP, and BBA contributed to the literature search, data extraction, review, and initial manuscript drafting. YRS, WAYM, KD, MGK, guided and supervised in different stages and contributed in the interpretation of data, revising the manuscript for important intellectual content and approval of the final manuscript.

All authors were involved in drafting and revising the manuscript and approved the final version.

## Consent for publication

Not applicable.

## Registration of research studies

1. Name of the registry: PROSPERO
2. Unique Identifying number or registration ID: CRD42021268800
3. Hyperlink to your specific registration (must be publicly accessible and will be checked): https://www.crd.york.ac.uk/prospero/display_record.php?RecordID=268800.

## Guarantor

Markos G. Kashiouris, MD MPH.

## Availability of data and materials

The data analyzed during the current study are available within manuscript and supplementary files.

## Provenance and peer review

Not commissioned, externally peer-reviewed.

## Declaration of competing interest

The authors declare that they have no competing interests.
