# Supplementary material for: Hypothermia versus normothermia after out-of-hospital cardiac arrest: A systematic review and meta-analysis of randomized controlled trials
Source: Ann Med Surg (Lond). 2022 Jan 29;74:103327. doi: 10.1016/j.amsu.2022.103327 (PMC8818536; doi:10.1016/j.amsu.2022.103327)
Supplement: Multimedia component 1 [file mmc1.docx]

Supplementary file 1. Electronic search details

| **Database** | Details, Search date: July 24, 2021 |
| --- | --- |
| **Embase** | **Search builder:** ('targeted temperature management' OR 'ttm' OR 'targeted hypothermia' OR 'targeted normothermia') AND ('out-of-hospital cardiac arrest' OR 'ohca' OR 'sudden cardiac arrest' OR 'sca')  **Result:** 913  **Link:** <https://www.embase.com/?phase=continueToApp#advancedSearch/resultspage/history.3/page.1/25.items/orderby.date/source>. |
| **Scopus** | **Search builder:** (((( targeted  AND temperature  AND management )  OR  ( ttm )  OR  ( targeted  AND hypothermia )  OR  ( targeted  AND normothermia ))  AND  (( out-of-hospital  AND cardiac  AND arrest )  OR  ( ohca )  OR  ( sudden  AND cardiac  AND arrest )  OR  ( sca ))))  **Result:** 633  **Link:** <https://www.scopus.com/results/results.uri?sid=b9bf4cd85fb8e07e5440d6e663a6533b&src=s&sot=b&sdt=b&origin=searchbasic&rr=&sl=198&s=TITLE-ABS-KEY((((targeted%20temperature%20management)%20OR%20(TTM)%20OR%20(targeted%20hypothermia)%20OR%20(targeted%20normothermia))%20AND%20((out-of-hospital%20cardiac%20arrest)OR%20(OHCA)%20OR%20(Sudden%20cardiac%20arrest)%20OR%20(SCA))))&searchterm1=(((targeted%20temperature%20management)%20OR%20(TTM)%20OR%20(targeted%20hypothermia)%20OR%20(targeted%20normothermia))%20AND%20((out-of-hospital%20cardiac%20arrest)OR%20(OHCA)%20OR%20(Sudden%20cardiac%20arrest)%20OR%20(SCA)))&searchTerms=&connectors=&field1=TITLE_ABS_KEY&fields>= |
| **PubMed Central** | **Search:** ((targeted temperature management) OR (TTM) OR (targeted hypothermia) OR (targeted normothermia)) AND ((out of hospital cardiac arrest) OR (ohca) OR (sudden cardiac arrest) OR (SCA))  **Link:** <https://www.ncbi.nlm.nih.gov/pmc/?term=((+targeted+temperature+management)+OR+(TTM)+OR+(targeted+hypothermia)+OR+(targeted+normothermia))+AND+((out+of+hospital+cardiac+arrest)+OR+(ohca)+OR+(sudden+cardiac+arrest)+OR+(SCA))>  **Result:** 9508 |
| **PubMed** | **Search:** ((targeted temperature management) OR (TTM) OR (targeted hypothermia) OR (targeted normothermia)) AND ((out-of-hospital cardiac arrest) OR (OHCA) OR (Sudden cardiac arrest) OR (SCA))  **Link:** <https://pubmed.ncbi.nlm.nih.gov/?term=%28%28targeted+temperature+management%29+OR+%28TTM%29+OR+%28targeted+hypothermia%29+OR+%28targeted+normothermia%29%29+AND+%28%28out-of-hospital+cardiac+arrest%29OR+%28OHCA%29+OR+%28Sudden+cardiac+arrest%29+OR+%28SCA%29%29&filter=years.2000-2021&size=200>  **Result:** 1845 |
