# Supplementary material for: Hypothermia versus normothermia after out-of-hospital cardiac arrest: A systematic review and meta-analysis of randomized controlled trials
Source: Ann Med Surg (Lond). 2022 Jan 29;74:103327. doi: 10.1016/j.amsu.2022.103327 (PMC8818536; doi:10.1016/j.amsu.2022.103327)
Supplement: Multimedia component 2 [file mmc2.docx]

Supplementary file 2:

Table. Basic details of included studies

| ID | Title | Country where study was conducted | Study design | Start date | End date | Inclusion criteria | Exclusion criteria | Other outcomes |
| --- | --- | --- | --- | --- | --- | --- | --- | --- |
| Bernard et al, 2002 | Treatment of comatose survivors of out-of-hospital cardiac arrest with induced hypothermia | Australia | RCT | September 1996 | June 1999 | Initial cardiac rhythm of ventricular fibrillation at the time of arrival of ambulance, successful return of spontaneous circulation, persistent coma after return of spontaneous circulation, transfer to one of four participating emergency departments | Age of less than 18 years for men, age of less than 50 years for women (because of possibility of pregnancy), cardiogenic shock (systolic blood pressure of less than 90 mmHg despite epinephrine infusion), or possible causes of coma other than cardiac arrest (drug overdose, head trauma, or cerebrovascular accident), intensive care bed not available at the participating institution |  |
| Dankiewicz et al, 2021 | Hypothermia versus Normothermia after Out-of-Hospital Cardiac Arrest | Sweden | RCT | November 2017 | January 2020 | Out-of-hospital cardiac arrest of a presumed cardiac or unknown cause, Sustained Return of spontaneous circulation (ROSC) - defined as 20 minutes with signs of circulation without the need for chest compressions, Unconsciousness defined as not being able to obey verbal commands (FOUR-score motor response of <4) and no verbal response to pain after sustained ROSC, Eligible for intensive care without restrictions or limitations, Inclusion within 180 minutes of ROSC | Unwitnessed cardiac arrest with an initial rhythm of asystole, Temperature on admission <30°C, On Extracorporeal Membrane Oxygenation prior to ROSC, Obvious or suspected pregnancy, Intracranial bleeding, Severe chronic obstructive pulmonary disorder (COPD) with long-term home oxygen therapy |  |
| Hachimi-Idrissi et al, 2004 | The evolution of serum astroglial S-100 β protein in patients with cardiac arrest treated with mild hypothermia | Belgium | RCT | October 1999 | June 2002 | In the short study period (SSP) study of mild hypothermia, eligible patients were in asystole or in pulseless electrical activity, were older than 18 years of age and had a tympanic temperature of over 30 ◦C. They had a Glasgow coma scale<7, were not pregnant, had no known coagulopathy and no history of central nervous system depressant drug prior to CA. In the long study period (LSP) study of mild hypothermia, eligible patients were aged between 18 and 75 years, and had a witnessed cardiac arrest of cardiac origin with ventricular fibrillation (VF) or non-perfusing ventricular tachycardia (VT) with had an estimated interval of 5–15 min from collapse to first attempt at resuscitation by emergency medical personnel, and an interval of <60 min from collapse to ROSC. | Exclusion criteria included patients with CA resulting from intoxication or trauma, responding to verbal command after ROSC, with tympanic temperature <30 ◦C at admission, evidence of hypotension (mean arterial pressure <60 mmHg for more than 30 min on admission), terminal illness, preexisting coagulopathy, pregnancy and unavailability for follow up. | Days from randomization to hospital discharge (median (IQR)) Overall T = 9.4 (4.0–17.0) C = 9.8 (5.0–17.4)[ among Survivors T= 15.4 (10.4–25.4) C= 14.6 (9.7–23.6); Died in hospital T= 4.0 (2.0–7.0) C = 5.0 (2.0–8.0)]; Days from randomization to ICU-discharge(median (IQR)) [Overall T = 4.9 (3.0-8.3) C= 4.8 (2.9-8.0); Survivors T= 5.9 (3.9–9.6) C = 5.4 (3.2–8.9); Died in the ICU T = 3.8 (1.2–5.8) C =3.9 (1.4–6.2)] Days from randomization to extubation or death (n=1759)(median (IQR)) [ Overall T =3.7 (1.9–6.0) C = 3.2 (1.8–5.7) Survivors T = 3.8 (2.0–6.4) C = 2.9 (1.9–5.5); **Poor functional outcome** at 6 mo, T= 495/918, C= 493/911; **Health-related quality of life** at six months: All participants* Median score: T= 0(IQR: 0 – 80) C=Median score: 0 (IQR: 0 – 80); Participants alive at six months , T=Mean score: 74+-20 C=75+-20; **Adverse Events:** Shivering on Day 1, None: T= 673/883 C=798/884, Mild T=75/883 C= 37/884 Moderate T=95/883 C=36/884; Severe T= 40/883 C= 13/884, Shivering on Day 2: None, T= 618/847 C= 720/864 , Mild: T= 93/847 C= 59/864, Moderate: T=103/847 C= 58/864, Severe T=33/847 C=27/864; Shivering on Day 3: None:T= 606/793 C= 650/793 Mild: T=74/793, C= 49/793, Moderate: T=77/793 C= 60/793, Severe: T=36/793 C= 34/793; **Serious adverse events** — Arrhythmia resulting in hemodynamic compromise , T= 222/927, C= 152/921; Bleeding T=44/927, C= 46/922; Skin complication related to device used for targeted temperature management , T=10/927, C= 5/922; Pneumonia, T= 330/927 C= 322/921; Sepsis , T=99/926, C= 83/922 |
| Hypothermia after Cardiac Arrest Study Group, 2002 | Mild therapeutic hypothermia to improve the neurologic outcome after cardiac arrest | Austria, Belgium, Finland, Germany, and Italy | RCT | March 1996 | January 2001 | ROSC after a witnessed cardiac arrest, ventricular fibrillation or nonperfusing ventricular tachycardia as the initial cardiac rhythm, a presumed cardiac origin of the arrest, an age of 18 to 75 years, an estimated interval of 5 to 15 minutes from the patient’s collapse to the first attempt at resuscitation by emergency medical personnel, and an interval of no more than 60 minutes from collapse to restoration of spontaneous circulation | Tympanic-membrane temperature below 30°C on admission, a comatose state before the cardiac arrest due to the administration of drugs that depress the central nervous system, pregnancy, response to verbal commands after the return of spontaneous circulation and before randomization, evidence of hypotension (mean arterial pressure, less than 60 mm Hg) for more than 30 minutes after the return of spontaneous circulation and before randomization, evidence of hypoxemia (arterial oxygen saturation, less than 85 percent) for more than 15 minutes after the return of spontaneous circulation and before randomization, a terminal illness that preceded the arrest, factors that made participation in follow-up unlikely, enrollment in another study, the occurrence of cardiac arrest after the arrival of emergency medical personnel, or a known preexisting coagulopathy | Complications T=98/135 C=93/132; Bleeding of any severity T=35/135 C=26/138; Need for platelet transfusion T=2/135 C=0/138; Pneumonia T=50/135 C=40/137; Sepsis T=17/135 C=9/138; Pancreatitis T=1/135 C=2/138; Renal failure T=13/135 C=14/138; Hemodialysis T=6/135 C=6/138; Pulmonary edema T=9/136 C=5/133; Seizures T=10/136 C=11/133; Lethal or long-lasting arrythmia T=49/135 C=44/138; Pressure sores T=0/136 C=0/133 |
| Laurent et al, 2005 | High-volume hemofiltration after out-of-hospital cardiac arrest: a randomized study | France | RCT | May 2000 | March 2002 | Patients between age 18 to 75 who had a cardiac arrest with ventricular fibrillation or asystole, interval of less than 10 minutes to CPR and ROSC within 50 minutes of initiation of CPR were included | Pregnancy, response to verbal commands after ROSC, or a terminal illness present before the cardiac arrest | Hospital mortality: T=12/22; C=11/20; VT in first 24 hours in ICU: T=6/22 C=2/20 |
| Nielsen et al,2013 | Targeted temperature management at 33°C versus 36°C after cardiac arrest | Europe and Australia | RCT | November 2010 | January 2013 | Patients 18 years or older who were admitted with out of hospital cardiac arrest of presumed cardiac cause and had GCS less than 8 were screened. Patients with 20 or more consecutive minutes of spontaneous circulation were included. | An interval from the return of spontaneous circulation to screening of more than 240 minutes, unwitnessed arrest with asystole as the initial rhythm, suspected or known acute intracranial hemorrhage or stroke, and a body temperature of less than 30°C | Serious adverse events: T:439/472; C:417/464 |
