# Supplementary material for: Hypothermia versus normothermia after out-of-hospital cardiac arrest: A systematic review and meta-analysis of randomized controlled trials
Source: Ann Med Surg (Lond). 2022 Jan 29;74:103327. doi: 10.1016/j.amsu.2022.103327 (PMC8818536; doi:10.1016/j.amsu.2022.103327)
Supplement: Multimedia component 3 [file mmc3.docx]

**Supplementary file 3. Additional analysis**

**Mortality till 6 months follow up**

Excluding studies with sample less than 50 in each arm


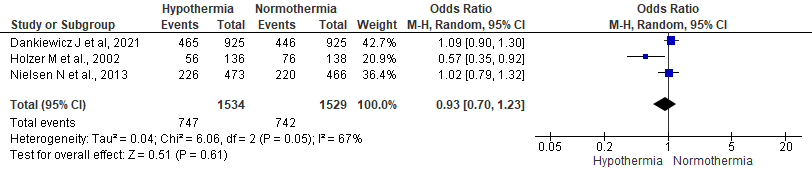


**Figure 1.** Forest plot comparing six month mortality across hypothermia and normothermia protocol using random effect model excluding studies with sample less than 50 in each arm

Excluding all the studies published before 2010 and pooling data from Nielsen N et al., and Dankiewicz J et al using fixed effect model


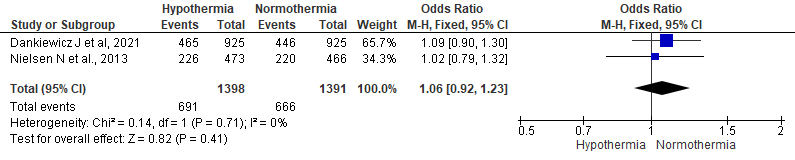


**Figure 2.** Forest plot comparing six month mortality across hypothermia and normothermia protocol using random effect model excluding all the studies published before 2010

**Unfavorable neurological outcomes:**

Unfavorable neurological outcome with CPC 3 or 4, or mRS of 4 or 5 using fixed effect model


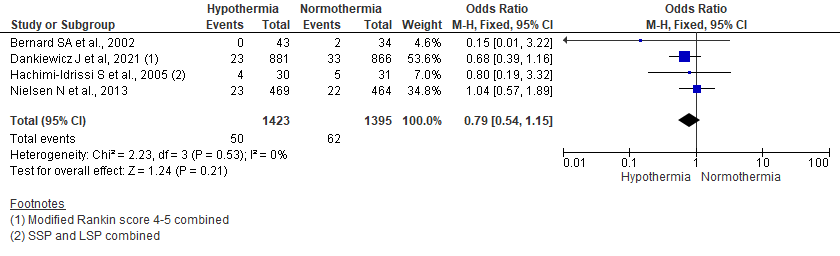


**Figure 3.** Forest plot comparing unfavorable neurological outcome six month following OHCA across hypothermia and normothermia protocol using fixed effect model
